# Androgen receptor‐neuroendocrine double‐negative tumor with squamous differentiation arising from treatment‐refractory metastatic castration‐resistant prostate cancer

**DOI:** 10.1002/iju5.12363

**Published:** 2021-08-22

**Authors:** Harutake Sawazaki, Atsushi Asano, Yosuke Kitamura, Jumpei Katsuta, Yuji Ito

**Affiliations:** ^1^ Department of Urology Tama‐Hokubu Medical Center Higashimurayama Japan; ^2^ Department of Urology National Defense Medical College Tokorozawa Japan; ^3^ Department of Pathology Tama‐Hokubu Medical Center Higashimurayama Japan

**Keywords:** androgen receptor, double‐negative tumor, metastatic castration‐resistant prostate cancer, neuroendocrine tumor, squamous differentiation

## Abstract

**Introduction:**

Treatment‐refractory metastatic castration‐resistant prostate cancer is a heterogeneous disease classified into androgen receptor‐high prostate cancer, androgen receptor‐low prostate cancer, amphicrine prostate cancer co‐expressing androgen receptor and neuroendocrine genes, double‐negative prostate cancer lacking androgen receptor and neuroendocrine gene expression, and small cell or neuroendocrine prostate cancer without androgen receptor activity. Double‐negative tumors can convert to the squamous phenotype.

**Case presentation:**

A 62‐year‐old man was newly diagnosed with prostate cancer (serum prostate‐specific antigen 2613 ng/mL, Gleason score 4 + 5 = 9, cT3aN1M1b) that progressed to castration resistance 4 months after starting abiraterone with androgen deprivation therapy. After enzalutamide and docetaxel failed, a right ilium metastasis newly emerged. Needle biopsy confirmed a metastatic tumor with squamous differentiation that was CK5/6‐positive and chromogranin A‐, synaptophysin‐, and androgen receptor‐negative.

**Conclusion:**

We encountered a case of double‐negative prostate cancer with squamous differentiation identified by needle biopsy of a right ilium metastasis after abiraterone, enzalutamide, and docetaxel failure.

**Abbreviations & Acronyms**
AMPC: amphicrine prostate cancer composed of cells co‐expressing AR and NE genes
AR: androgen receptor
ARI: AR signaling inhibitor
ARLPC: AR‐low prostate cancer
ARPC: AR‐high prostate cancer
CGA: chromogranin A
CK5/6: cytokeratin5/6
CT: computed tomography
DNPC: double‐negative tumors lacking the expression of AR and NE genes
EP: etoposide and cisplatin
H&E: hematoxylin and eosin
LHRH: luteinizing hormone‐releasing hormone
mCRPC: metastatic castration‐resistant prostate cancer
NE: neuroendocrine
NEK6: mitotic‐related serine/threonine kinase
NSE: neuron‐specific enolase
PCa: prostate cancer
PDX: patient‐derived xenograft
PSA: prostate‐specific antigen
SCNPC: tumors with small cell or NE gene expression without AR activity
SYP: synaptophysin

**Keynote message**

We encountered a case of metastatic castration‐resistant prostate cancer in which needle biopsy of a right ilium metastasis revealed a double‐negative tumor with squamous differentiation after failure of abiraterone, enzalutamide, and docetaxel. He died 22 months after initial treatment. Further studies are needed to reveal the mechanisms of squamous transition of a double‐negative tumor and to establish a treatment strategy.

## Introduction

AR signaling is important in the development of PCa.^1^, ^2^ Metastatic castration‐sensitive PCa is typically treated by androgen deprivation therapy but progression to castration resistance is inevitable.^1^, ^2^ Therefore, sequential therapy using next‐generation ARIs such as enzalutamide or abiraterone and taxane‐based chemotherapy are administered. Prolonged AR‐signaling‐pathway inhibition induces AR‐independent clonal evolution, leading to treatment‐emergent NE carcinoma.^1^, ^2^, ^3^ Treatment‐refractory mCRPC is a heterogeneous disease with diverse drivers of progression and is categorized into five phenotypes based on the expression of AR or NE genes: ARPC, ARLPC, AMPC, DNPC lacking expression of AR and NE genes, and SCNPC with NE gene expression without AR activity.^2^ Although DNPC can convert to squamous phenotype,^2^ there are few reports of mCRPC with squamous differentiation.^4^ Herein, we describe a case of mCRPC in which DNPC with squamous differentiation was identified in a right ilium metastasis after abiraterone, enzalutamide, and docetaxel failure.

## Case presentation

**Fig. 1:**
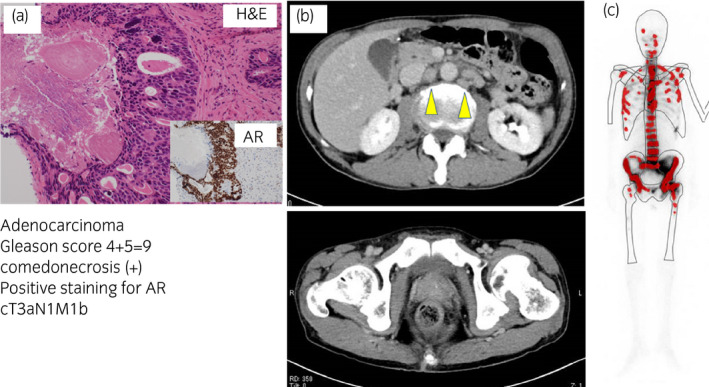
(a) Prostate needle biopsy revealed prostate cancer with a Gleason score 4 + 5=9 and comedonecrosis. Immunohistochemically, the tumor cells were positive for AR. (b) Computed tomography revealed multiple lymph node metastases without visceral metastasis (arrow head). (c) Bone scintigraphy revealed multiple bone metastases to the skull, scapula, spine, ribs, pelvis and femur (red dots). The clinical stage was T3aN1M1b.

**Fig. 2:**
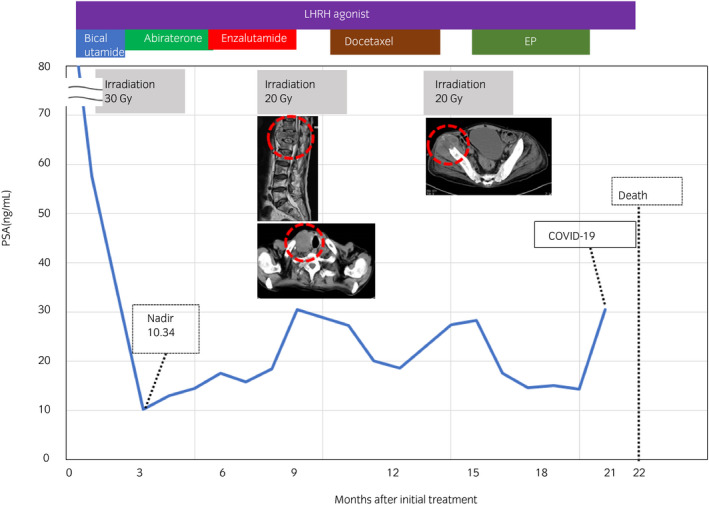
Treatment sequence, longitudinal changes in PSA, and outcomes. Metastatic castration‐sensitive prostate cancer progressed rapidly to metastatic castration‐resistant prostate cancer at 4 months after initial treatment. After treatment with enzalutamide, a metastatic tumor was found to be compressing the spinal cord at the T12 level and a right cervical lymph node metastasis (red dotted circle) was identified. A new metastasis in the right ilium (red dotted circle) emerged after docetaxel chemotherapy. EP chemotherapy was subsequently administered. The patient died 22 months after the initial treatment. [Correction added on 11 October 2021, after first online publication: “ng/dL” to “ng/mL” has been amended in the figure].

**Fig. 3:**
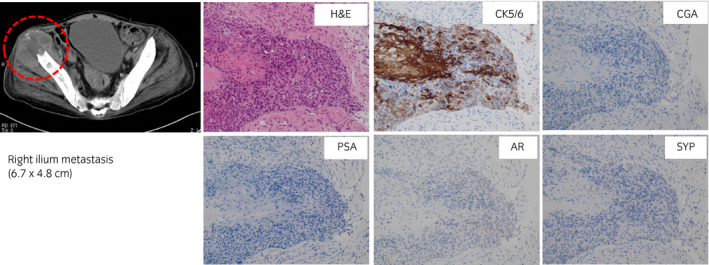
Computed tomography image showing a new metastatic lesion (red dashed circle) in the right ilium after 3 cycles of docetaxel. Microscopic analysis of a needle biopsy specimen taken from this site confirmed metastatic prostate carcinoma with squamous differentiation. Immunohistochemically, the tumor cells were positive for CK5/6 and negative for PSA, CGA, SYP, and AR.

A 62‐year‐old Japanese man with no significant past medical or family history was referred to our hospital for investigation of appetite loss and back pain. Serum PSA level was 2,613 ng/mL. Needle biopsy revealed a histological diagnosis of PCa with Gleason score 4 + 5 = 9 and comedonecrosis (Fig. 1a). Multiple lymph node metastases without visceral metastasis were identified on CT (Fig. 1b). Bone scintigraphy revealed multiple bone metastases (Fig. 1c). The sequence of treatment and longitudinal changes in PSA are summarized in Fig. 2. He was treated first with bicalutamide (80 mg/day) and leuprolide. Metastasis to the hip bone was locally irradiated (30 Gy) to relieve bone pain. One month later, he was treated with abiraterone (1000 mg/day) plus prednisolone (5 mg/day). PSA level decreased to a nadir of 10.34 ng/mL, but subsequently increased steadily to 17.57 ng/mL. Abiraterone was changed to enzalutamide (160 mg/day), but PSA levels increased to 30.55 ng/mL. Three months after starting enzalutamide, he reported numbness in the lower extremities, and magnetic resonance imaging revealed a metastatic tumor compressing the spinal cord at T12. A new right cervical lymph node metastasis was also seen on CT. Although the T12 metastasis was locally irradiated (20 Gy), he developed complete paralysis below the waist. Docetaxel (70 mg/m^2^) was subsequently administered every 4 weeks and serum PSA decreased to 18.68 ng/mL after three cycles. However, CT revealed progression of the T12 and right cervical lymph node metastases as well as a new metastasis (6.7 × 4.8 cm) in the right ilium. Microscopic analysis of needle biopsy specimen from the new metastasis confirmed metastatic tumor with squamous differentiation. On immunohistochemistry, tumor cells were positive for CK5/6 and negative for PSA, chromogranin A, synaptophysin, and AR (Fig. 3). Pathological diagnosis was DNPC with squamous differentiation. Despite irradiation, the right ilium and cervical lymph node metastases continued to grow. An elevated serum NSE level of 240 ng/mL was found. Combination chemotherapy consisting of etoposide 120 mg/day on days 1–3 and cisplatin 100 mg/day on day 1 (EP) was started. After one course of EP chemotherapy, CT revealed shrinkage of the T12, right ilium, and right cervical lymph node metastases. Serum NSE level decreased to 25.5 ng/mL. After three courses of EP therapy, the patient contracted COVID‐19 and could not receive further chemotherapy. His general condition worsened and serum NSE level increased to 452 ng/mL. He died 22 months after initial treatment. [Correction added on 13 October 2021, after first online publication: “ng/dL” to “ng/mL” has been amended in the Case Presentation section].

## Discussion

Recent studies have reported AR‐signaling loss in a substantial number of ARI‐refractory mCRPCs. Increased use of ARI therapy is associated with an increase in treatment‐refractory CRPC metastases having AR‐null phenotypes, including tumors with NE carcinoma or DNPC.^2^ Labrecque *et al*. reported a series of 55 patients with 98 treatment‐refractory mCRPC tumors detected at rapid autopsy. Data from these patients, a PDX model, and cell lines suggested that ARPC can transition to ARLPC, AMPC, DNPC, and SCNPC, circumventing treatments that suppress the hormone or AR pathway. Squamous pearl structures were identified in DNPC tissues from the patients and PDX models. DNPC is a proliferative AR‐null intermediate with inherent plasticity and the potential to convert to SCNPC or undergo squamous differentiation.^2^ In our case, a new metastasis emerged in the right ilium after treatment with abiraterone, enzalutamide, and docetaxel. AR‐null phenotypes may convert to squamous differentiation.

The mechanisms by which DNPC undergoes squamous transition remain uncertain.^2^ Lung adenocarcinoma can transition to squamous cell carcinoma through LKB1 loss, but there is no evidence of LKB1/STK11 loss in patients with mCRPC.^2^ Choudhury *et al*. identified a mitotic‐related serine/threonine kinase (NEK6) that mediates androgen‐independent tumor growth in PCa. In a mouse xenograft model, NEK‐mediated androgen‐independent tumors were primarily squamous in histology and AR‐negative. NEK‐mediated castration resistance did not require AR activity. Trans‐differentiation to the squamous phenotype occurred in response to castration.^5^ Francis *et al*. reported that β‐catenin promoted formation of squamous epithelia during growth of the prostate gland, even in the absence of androgen in vivo. Overexpression of β‐catenin interacted with PTEN loss to form highly invasive prostate carcinomas and squamous metaplasia.^6^

There is no standard treatment strategy for DNPC.^2^ DNPC‐specific immune‐related genes such as interleukin‐8 and CXCR1 are reported to promote metastasis of CRPC and angiogenesis. Furthermore, genes such as TGFβ and RUNX2 support tumor growth in bone. Upregulated genes common to ARLPC and AR‐null phenotypes are enriched in immune, inflammatory, and defense responses. Therefore, DNPCs may be responsive to immunotherapy.^2^ In our case, irradiation was ineffective for the new metastasis in the right ilium, but this metastasis decreased in size after treatment with EP. Although no component of small cell or NE carcinoma was identified in the biopsy specimen of the right ilium metastasis, we found an elevated serum NSE level. We considered it possible that a component of small cell or NE carcinoma was converted from AR‐null phenotypes present outside the biopsy site, and thus EP therapy may be effective for this component.

## Conclusion

We encountered a case of mCRPC in which needle biopsy of a right ilium metastasis revealed a double‐negative tumor with squamous differentiation after abiraterone, enzalutamide, and docetaxel failure.

## Conflict of interest

The authors declare no conflict of interest.

## Approval of the research protocol by an institutional reviewer board

The study was approved by the relevant ethics committee.

## Informed consent

Written informed consent was obtained from the patient’s family.

## Registry and the registration no. of the study/trial

Not applicable.
